# ADME SARfari: comparative genomics of drug metabolizing systems

**DOI:** 10.1093/bioinformatics/btv010

**Published:** 2015-01-08

**Authors:** Mark Davies, Nathan Dedman, Anne Hersey, George Papadatos, Matthew D. Hall, Lourdes Cucurull-Sanchez, Phil Jeffrey, Samiul Hasan, Peter J. Eddershaw, John P. Overington

**Affiliations:** ^1^European Molecular Biology Laboratory, European Bioinformatics Institute (EMBL-EBI), Wellcome Trust Genome Campus, Hinxton, Cambridge CB10 1SD, ^2^GlaxoSmithKline R&D, Gunnels Wood Road, Stevenage SG1 2NY and ^3^Pfizer Ltd., Granta Park, Great Abington, Cambridge CB21 6GP, UK

## Abstract

**Motivation:** ADME SARfari is a freely available web resource that enables comparative analyses of drug-disposition genes. It does so by integrating a number of publicly available data sources, which have subsequently been used to build data mining services, predictive tools and visualizations for drug metabolism researchers. The data include the interactions of small molecules with ADME (absorption, distribution, metabolism and excretion) proteins responsible for the metabolism and transport of molecules; available pharmacokinetic (PK) data; protein sequences of ADME-related molecular targets for pre-clinical model species and human; alignments of the orthologues including information on known SNPs (Single Nucleotide Polymorphism) and information on the tissue distribution of these proteins. In addition, *in silico* models have been developed, which enable users to predict which ADME relevant protein targets a novel compound is likely to interact with.

**Availability and implementation:** https://www.ebi.ac.uk/chembl/admesarfari

**Contact:** jpo@ebi.ac.uk

**Supplementary information**: Supplementary data are available at *Bioinformatics* online.

## 1 Introduction

The aim of drug discovery is to design and deliver a potential therapeutic agent, not only efficacious for the chosen disease but also with an acceptable dosage and safety profile. It is important to understand how a drug's disposition and its interaction with drug-metabolizing enzymes and transporters in pre-clinical species are likely to translate to humans. This could contribute to a more refined means of predicting human systemic exposure and any adverse events such as drug–drug interactions or tissue-specific toxicities arising from the species-specific disposition of the drug molecule and/or its metabolites.

There are a large number of examples where expensive clinical drug development programs had to be terminated due to an unfavourable PK profile. For example, in the literature, there is evidence on several drug candidates that are metabolized by aldehyde oxidase (AOX1) (e.g. Austin *et al*., 2001, Hutzler *et al*., 2013) where large species differences in gene structure and regulation have led to wide variance of exposure (and consequently efficacy and safety) between species.

Currently, identifying and integrating ADME (absorption, distribution, metabolism and excretion) -related data in a consistent way to aid species PK investigations is not a trivial process. For this reason, we have developed a new computational system called ADME SARfari, which integrates, genetic, proteomic, phenotypic and molecule interaction data and offers the potential of becoming a resource for community ADME data exchange and re-use. The primary data source for interaction and pharmacokinetic (PK) data is the ChEMBL database (Bento *et al*., 2014). To broaden the scope and analytical power of ADME SARfari, we have included data from resources such as PharmaADME (http://www.pharmaadme.org), Ensembl (Flicek *et al.,* 2014) and the Human Protein Atlas (Uhlen *et al*., 2010). To access the data, a freely accessible web interface has been developed, which presents users with a summary of ADME and PK data related to protein targets and compounds relevant to their specific field of research.

## 2 Methods

### 2.1 Data collection

The PharmaADME core and extended gene lists were used as the primary source for the list of ADME-related genes. Further processing of the ADME gene list, which included the removal of pseudogenes and duplicates and the inclusion of additional ADME-related genes, resulted in a final list of 303 human ADME genes. An automated process using the EnsemblCompara API (Vilella *et al.*, 2009) added orthologous genes from model organisms to the list. The final collection of ADME genes was mapped to the ChEMBL database, which allowed the ADME-related bioactivity and compound data to be extracted.

The orthologue mapping was extended to include a mapping between the predicted protein sequences found within the *Sus scrofa*, Goettingen minipig and the *Canis familiaris* beagle genomes (Vamathevan *et al*., 2013). The reason for including these relatively new specific genomes is their popularity in preclinical toxicology studies and known intra-strain variation of PK (Bode *et al*., 2010).

The Human Protein Atlas resource was used as the reference source of protein expression levels in various human cell types and tissues.

### 2.2 Model building and validation

In an attempt to bridge the chemistry/biology/ADME space, predictive models for suitable ADME genes were generated and validated. When given a chemical structure as input, these models return a ranked list of likely ADME targets based on the structural similarity to other compounds and activity trends within ADME SARfari. This is described in more detail in the Supplementary Information S1.

### 2.3 Database and interface

The data described in Section 2.1 was loaded into an Oracle 11g database, where it was then further enriched. One such example of the data enrichment is the generation of multiple sequence alignments of the groups of orthologues. Access to the data is provided via a Web-based application, which provides users with a client-side feature-rich interface. Much of the functionality the web application relies upon is built into a series of RESTful web services.

## 3 Results

The ADME SARfari interface presents the user with a series of sections, which correspond to the different datasets parsed and loaded into the ADME SARfari system. The different sections are *Homepage, Orthologues, Tissues, Bioactivities, Molecules, Pharmacokinetics* and *About*. As well as being able to generate complex workflows, which can help answer ADME-related questions, the user is free to browse and export data from the system’s various sections, without needing to run a pre-emptive search.

### 3.1 Workflows and research competency questions

To outline the functionality offered by the ADME SARfari system, a sequence of industry-focused ADME research scenarios and their corresponding ADME SARfari workflows are shown in Supplementary Table S1.

### 3.2 Orthologue summary

The orthologue summary page (see Supplementary Fig. S1B) presents a tabular overview of all ADME-related genes. Each cell within the first column of the table corresponds to the unique set of human proteins and the subsequent columns contain proteins from potential model organisms. This results in each row of the table representing a set of orthologues. The page provides a number of data manipulation tools including options to filter, sort and toggle the display of columns. The orthologue summary page also presents the user with search context-specific colouring. For example, if a user conducts a BLAST search, they will be directed to the orthologue summary page and only rows that contain at least one BLAST ‘hit’ are returned (a hit is defined as any ADME protein sequence, which shares sequence identity >35% to the user submitted query). To help interpret the BLAST results, each protein target ‘lozenge’ is coloured using a dark to light-green colouring scheme, where darker shades of green indicate higher sequence identity to the user query. Similarly, when a user conducts a model prediction-based search, only rows that contain at least one gene product predicted to interact with user submitted smaller molecule are returned. The model prediction hits can be identified as those protein target ‘lozenges’ that are coloured green.

### 3.3 ADME target prediction model

The target prediction model (Supplementary Information S1) is exposed to end users *via* the web interface. The service allows users to draw molecules using the ChemAxon Marvin JS chemical sketcher (https://www.chemaxon.com/products/marvin/marvin-js/) or upload structures, which can then be submitted to the predictive model. The results of the search are displayed in the Orthologue summary page. Here, the top 10 targets predicted to interact with user-submitted molecule are highlighted in green.

### 3.4 Pharmacokinetic summary

To provide a cross-species comparison of PK measurements, the Pharmacokinetic summary page was created (see Supplementary Fig. S1C). This allows a user to compare normalized PK measurement values [clearance (*CL*), maximum plasma concentration (*C*_max_), bioavailability (*F*), plasma half life (*T*_1/2_), time for maximum plasma concentration (*T*_max_) and volume of distribution (*V*_d_)]. This is carried out by binning the PK measurement values into low (dark red), medium (red) and high (light red) ranges. Associating each range with a colour allows a user to visually compare inter-species PK measurements.

## 4 Further work

The ADME SARfari system is a novel and completely open web-based tool, aimed to assist researchers working on translational ADME studies. Future technical improvements include automating access, by exposing the underlying RESTful Application Programming Interface. This will allow developers and users familiar with workflow tools, such as KNIME and Pipeline Pilot, to reuse these components and extend the Use Cases defined in Supplementary Table S1.

We see the system as a tool to assist users in the selection and design of costly preclinical ADME/PK assays and subsequent interpreting the results of these assays. An underlying constraint imposed on the system is the dependence on published ADME data. To help enhance all actual (e.g. orthologue mappings, tissue expression levels), predicted (e.g. ADME protein interaction models) and inferred (e.g. cross-species PK comparison) results produced by the system, the subsequent direction is to identify additional sources of ADME data. We, therefore, encourage the ADME research community to contact us and consider depositing their datasets into ADME SARfari and/or the ChEMBL database.

## Supplementary Material

Supplementary Data
