# Supplementary material for: ADME SARfari: comparative genomics of drug metabolizing systems
Source: Bioinformatics. 2015 Jan 8;31(10):1695–7. doi: 10.1093/bioinformatics/btv010 (PMC4426839; doi:10.1093/bioinformatics/btv010)
Supplement: Supplementary Data [file supp_31_10_1695__index.html]

ADME SARfari: comparative genomics of drug metabolizing systems — ADME SARfari: comparative genomics of drug metabolizing systems — Supplementary Data 

# ADME SARfari: comparative genomics of drug metabolizing systems

## Supplementary Data

files

**Files in this Data Supplement:**

- Supplementary Data - docx file
- Supplementary Data - docx file
- Supplementary Data - doc file
