# Supplementary material for: ADME SARfari: comparative genomics of drug metabolizing systems
Source: Bioinformatics. 2015 Jan 8;31(10):1695–7. doi: 10.1093/bioinformatics/btv010 (PMC4426839; doi:10.1093/bioinformatics/btv010)
Supplement: Supplementary Data [file supp_btv010_ADME_SARfari_Supplementary_Figure_1.docx]

**Supplementary Figure 1**


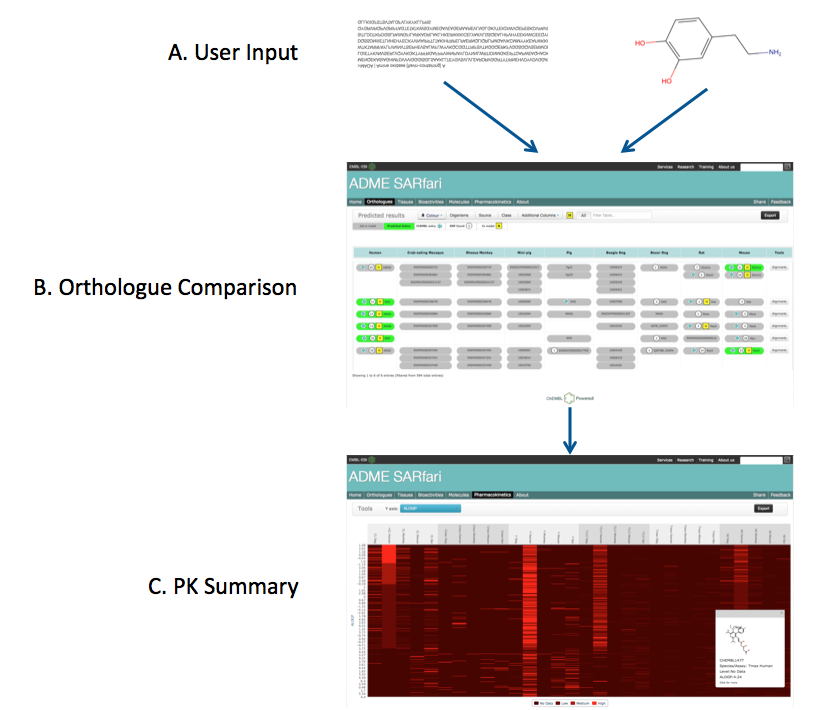


**Supplementary Figure 1.** Example ADME SARfari workflow.

A. User can search the system with a molecule or gene/protein query. B. The system will display gene products from human and model organism species in the form of an Orthologue Comparison matrix. C. The system presents a low/medium/high colour-grouped representation of PK measurements for molecules in a users search result set. The PK Summary representation makes it possible to compare a molecules PK measurements across human and model organism species.
