# Supplementary material for: ADME SARfari: comparative genomics of drug metabolizing systems
Source: Bioinformatics. 2015 Jan 8;31(10):1695–7. doi: 10.1093/bioinformatics/btv010 (PMC4426839; doi:10.1093/bioinformatics/btv010)
Supplement: Supplementary Data [file supp_btv010_ADME_SARfari_Supplementary_Information_1.docx]

**Supplementary Information 1**

Model Building and Validation

The methodology employed to build the ADME models was a multi-class Naive Bayes classifier (MCNBC) based on a multivariate Bernoulli model; an approach, which has been successfully used in previous ligand-based target prediction efforts (Nidhi *et al*., 2006, Koutsoukas *et al*., 2011, Chen *et al*., 2007). The MCNBC model was built using the carefully curated and filtered structural and bioactivity information from ChEMBL (version 17). In brief, the classifier learns the various categories (in this case ADME genes) by considering the frequency of occurrence of substructural features of active compounds for each of the categories/targets. Given a new, unseen compound, the model calculates a Bayesian probability score based on the molecule's individual features and produces a ranked list of probable targets. The model was built in Python using scikit-learn machine learning library (Pedregosa *et al*., 2011) and Morgan fingerprints calculated with the RDKit toolkit (<http://www.rdkit.org/>). Further details on the MCNBC model and specific implementation can be found elsewhere (Martinez-Jimenez *et al*., 2013).

A validation step was carried out to ensure that the ADME SARfari predictive model returned results with acceptable confidence. The first step is the model building stage, where the input data (ADME focused bioactivity data associating targets to molecules), was divided into a training set (85% of data) and a validation set (remaining 15% of data) making sure that there is no compound overlap between the two sets. Model validation was carried out by submitting molecules from the validation set, encoded as RDKit Morgan fingerprints, to the model built using only the training set data. A comparison of the rank position of the known target the molecule interacts with to the predicted target was then performed. This resulted in a cumulative rank recall of >70%, when taking into account the first 5 rank positions. Summary information and charts concerning the predictive model and the validation step are available on the *About* page of the ADME SARfari system.

References

Chen,B. *et al*. (2007) Evaluation of machine-learning methods for ligand-based virtual screening. *J. Comput. Aided Mol. Des.,* 21, 53–62.

Koutsoukas,A. *et al*. (2011) From in silico target prediction to multi-target drug design: current databases, methods and applications. *J. Proteomics* 74, 2554–2574

Martinez-Jimenez,F. et al. (2013) Target Prediction for an Open Access Set of Compounds Active against Mycobacterium tuberculosis. *PLoS Comput. Biol*., 9, e1003253.

Nidhi,G.M. *et al*. (2006) Prediction of biological targets for compounds using multiple-category Bayesian models trained on chemogenomics databases. *J. Chem. Inf. Model*., 46, 1124–1133.

Pedregosa,F. *et al*. (2011) Scikit-learn: Machine Learning in Python. *J. Mach. Learn. Res.*, 12, 2825–2830.
