# Supplementary material for: ADME SARfari: comparative genomics of drug metabolizing systems
Source: Bioinformatics. 2015 Jan 8;31(10):1695–7. doi: 10.1093/bioinformatics/btv010 (PMC4426839; doi:10.1093/bioinformatics/btv010)
Supplement: Supplementary Data [file supp_btv010_ADME_SARfari_Supplementary_Table_1.doc]

**Supplementary Table 1**

| **Drug Discovery Research Scenario** | **ADME SARfari Workflow** |
| --- | --- |
| **1.** My lead compound has a desirable in vitro binding profile to my novel disease target. Before I begin investing in expensive in vivo experiments, I wish to know if others have progressed similar compounds in vivo (in any species) and what the ADME route of these compounds have been like. This would help me highlight probability of success early on in my studies. | - Enter molecule structure on homepage and conduct ‘Similarity’ based search - Go to ‘Molecules’ page (default results page) and export data - Go to ‘Bioactivities’ page and export data |
| **2.** My department has identified a lead molecule for a certain disease indication. It has good PK in rat and I have some indications of its route of metabolism.  I wish to identify which  animal model that might be best suited to model human ADME. | - Go to ‘Orthologues’ page and search for the enzymes that the compound is metabolised by in rat. Use the sequence similarity to identify species with high similarity to rat and also human for the enzymes of interest. - Alternatively/in addition enter a molecular structure on the homepage and conduct a ‘Model Prediction’ search.  Use the predicted targets on the orthologues page to help guide the animal model selection. |
| **3.** I am working on a lead molecule and I would like to know if it is predicted to be metabolised by a known ADME metabolising enzyme. | - Enter molecule structure on homepage and conduct ‘Model Prediction’ search. - Go to ‘Orthologues’ page  (default results page), which displays ADME related proteins the molecule structure is predicted to bind to. |
| **4.** I am preparing a clinical study risk assessment document for a compound that has passed animal safety tests. I have good evidence that my compound interacts with 3 specific CYP enzymes. I want to highlight genetic polymorphisms in the human population that might cause unexpected ADME responses via interaction with this set of CYP enzymes. | - Conduct a protein target search for CYP enzymes (could use BLAST or Keyword) - Go to ‘Orthologues’ page (default results page), each row will correspond to a group of CYP orthologues - Click on ‘Alignments’ button at end of row to display multiple sequence alignment CYP orthologues. Amino acids highlighted with a circle are associated with SNP variation |
| **5.** As a consequence of Workflow 3, I want to report which human tissues are likely to be impacted by this variation. | - Following on from Workflow 4, go on ‘Tissues’ page - All tissue specific protein expression levels for targets returned by CYP based search will be on display |

**Supplementary Table 1.** Definitions of five drug discovery research scenarios and how they can be addressed using ADME SARfari.
